# Machine learning approach to predicting albuminuria in persons with type 2 diabetes: An analysis of the LOOK AHEAD Cohort

**DOI:** 10.1111/jch.14397

**Published:** 2021-11-30

**Authors:** Zeid Khitan, Tanmay Nath, Prasanna Santhanam

**Affiliations:** ^1^ Division of Nephrology Department of Medicine Joan C Edwards School of Medicine Marshall University Huntington West Virginia USA; ^2^ Department of Biostatistics Bloomberg School of Public Health Johns Hopkins University, Baltimore Maryland USA; ^3^ Division of Endocrinology Diabetes, & Metabolism Department of Medicine Johns Hopkins University School of Medicine Baltimore Maryland USA

**Keywords:** albuminuria, diabetes, machine learning, metabolic syndrome, proteinuria

## Abstract

Albuminuria and estimated glomerular filtration rate (e‐GFR) are early markers of renal disease and cardiovascular outcomes in persons with diabetes. Although body composition has been shown to predict systolic blood pressure, its application in predicting albuminuria is unknown. In this study, we have used machine learning methods to assess the risk of albuminuria in persons with diabetes using body composition and other determinants of metabolic health. This study is a comparative analysis of the different methods to predict albuminuria in persons with diabetes mellitus who are older than 40 years of age, using the LOOK AHEAD study cohort‐baseline characteristics. Age, different metrics of body composition, duration of diabetes, hemoglobin A1c, serum creatinine, serum triglycerides, serum cholesterol, serum HDL, serum LDL, maximum exercise capacity, systolic blood pressure, diastolic blood pressure, and the ankle‐brachial index are used as predictors of albuminuria. We used Area under the curve (AUC) as a metric to compare the classification results of different algorithms, and we show that AUC for the different models are as follows: Random forest classifier‐0.65, gradient boost classifier‐0.61, logistic regression‐0.66, support vector classifier ‐0.61, multilayer perceptron ‐0.67, and stacking classifier‐0.62. We used the Random forest model to show that the duration of diabetes, A1C, serum triglycerides, SBP, Maximum exercise Capacity, serum creatinine, subtotal lean mass, DBP, and subtotal fat mass are important features for the classification of albuminuria. In summary, when applied to metabolic imaging (using DXA), machine learning techniques offer unique insights into the risk factors that determine the development of albuminuria in diabetes.

## 1 INTRODUCTION

The estimated increase in diabetes prevalence is expected to post enormous burden on the health care resources affecting more than 400 million people between that age of 20–79 by the third decade of this century.^1^ Furthermore, among the different complications of diabetes, diabetes‐related chronic kidney disease (CKD) is of concern due to its gradual and indolent progression over several years, often culminating in renal replacement therapy.

Albuminuria and estimated glomerular filtration rate are early markers of future renal disease if employed promptly and to specific populations.^2^ In the past, diabetic nephropathy has been classified into microalbuminuria and macroalbuminuria.^3^ Additionally, poor glycemic blood control, increased blood pressure levels, and genetic factors have been identified as risks for diabetic nephropathy.^3^ Moreover, proteinuria, the cornerstone of diabetic nephropathy, can accelerate kidney disease progression to end‐stage renal disease (ESRD) through multiple pathways.^4^ Studies have also evaluated albuminuria in the context of worsening cardiac outcomes and have found it helpful independently and in combination with serum creatinine and e‐GFR.^5^, ^6^

Dual‐energy X‐ray absorptiometry (DXA) is an accurate and easy technique to quantify adipose tissue, muscle mass, and bone density in different compartments of the human body.^7^ However, although DXA measured body composition has been shown to predict systolic blood pressure, its application in predicting albuminuria is unknown.^8^ In this study, we have used machine learning methods to assess the different features that may predict albuminuria in persons with diabetes, using body composition and other widely employed determinants of vascular health.^9^

### 1.1 Research design, data, and methods

This study was a comparative analysis of the different machine learning methods to predict the presence of microalbuminuria/overt proteinuria in persons with diabetes mellitus older than 40 years, using the LOOK AHEAD study cohort (an NIH funded study‐ ClinicalTrials.gov Identifier: NCT00000620) baseline characteristics.^10^, ^11^, ^12^ The original study was performed at multiple different locations. We obtained the de‐identified data from the NIH‐NIDDK repository after obtaining IRB approval from the Johns Hopkins IRB.

The key aims of the study are (1) examination of the utility of body fat distribution in the prediction of albuminuria; (2) compare the different machine learning methods; (3) elucidate the critical determinants of albuminuria when analyzed by the random forest classifier.^13^

### 1.2 LOOK AHEAD study cohort

The LOOK AHEAD study had two groups. The intensive lifestyle intervention group achieved weight loss through dietary changes and increased physical activity, and a control group that received only diabetes support and education.^14^ The intervention group received individual and group sessions every week during the trial, while the control group received the usual care involving diet and education. In addition, persons with Type 2 Diabetes who met the following inclusion criteria were part of the study: (1) Age between 45 and 75; (2) Overweight or Obese status (BMI 25 kg/m^2^ or more, or 27 kg/m^2^ or more while on insulin); (3) blood pressure (BP) 160/100 mmHg or below; and (4) plasma triglyceride below 600 mg/dL.^10^, ^11^, ^12^ The inclusion and exclusion criteria can be found in these original manuscripts from the LOOK AHEAD group.^10^, ^11^, ^12^

### 1.3 Measurement of lipid values and A1C

Lipid parameters (total cholesterol, HDL‐cholesterol, LDL‐cholesterol, and triglycerides) were measured at the Look AHEAD Central Laboratory at Baseline annually for the first few years and every two years, during extended follow‐up period of the study. The levels were measured using standard methods previously described.^12^, ^15^ Ion exchange, high‐performance liquid chromatography was used to measure the A1C (Bio‐rad Variant,11).^11^, ^12^

### 1.4 Measurement of albuminuria

As per the original protocol, albumin and creatinine were measured (by the Look AHEAD Central Laboratory) in a spot urine sample at Baseline and annually through Year 4. Serum creatinine was also measured, and GFR was estimated. The albumin to creatinine ratio (ACR) was categorized into Normoalbuminuric (< 0.030); Micro‐albuminuria (0.030 ‐ 0.29); Overt Proteinuria (> = 0 .3) in the original Look Ahead study.^11^, ^12^ We classified the ACR as (1) Presence of albuminuria (Yes ‐combining the overt proteinuria and microalbuminuria) and (2) Absence of albuminuria (No ‐Normoalbuminuria)

### 1.5 Measurement of body composition by DXA

Whole‐body composition DXA measurements on over 1200 participants using the Hologic Scanner had been obtained as a part of the original study. Look Ahead DXA Quality Assurance Center at the University of California—San Francisco reviewed and tabulated the DXA data. We obtained the baseline data for our analysis.^11^, ^16^

## 2 METHODS

Initially, in our analysis, there were 1373 subjects analyzed using 17 features. These features included Age(years), Subtotal lean mass (g), Subtotal fat mass, Total fat percentage, Truncal lean mass, Truncal fat mass, Duration of diabetes(years), Hemoglobin A1c(%), Serum creatinine(mg/dlL), Serum triglycerides(mg/dL), Serum cholesterol(mg/dL), Serum HDL(mg/dL), Serum LDL(mg/dL), Maximum exercise capacity, Systolic blood pressure(SBP)(mmHg), Diastolic blood pressure(DBP), and the Ankle‐brachial index(ABI). Additionally, subjects with any missing features were excluded from the analysis. After excluding such subjects (N = 43), we used the remaining 1330 subjects for our machine learning algorithms. Further, we removed features that are correlated to each other. We chose an arbitrary correlation coefficient (*r* = 0.6) as the threshold to remove the correlated features. Specifically, we removed the Total fat percentage, Serum LDL, Truncal lean mass, and Truncal fat mass and used the remaining 13 features for our analysis. After that, we split the dataset into training (70%) and testing (30%) datasets. Specifically, there were 931 subjects in training and 399 subjects in the testing dataset. However, the training dataset suffered from class imbalance. Class imbalance happens when one of the classes has a relatively smaller number of samples than the other classes. For instance, out of 931 subjects in the training dataset, there were only 164 subjects with albuminuria and 767 without albuminuria, resulting in an imbalanced dataset. Training a model on an imbalanced dataset can lead to biased learning classification. We performed a Synthetic Minority Oversampling Technique to balance the training dataset, where additional training samples are generated for the minority class, which helps balance the overall dataset.^17^ After balancing the training dataset, we had 767 subjects in both classes.

We first performed exploratory analysis that confirmed the non‐normal distribution of almost all of the variables using both the Shapiro‐Wilk and the Kolmogorov‐Smirnov test (all values were < 0.01‐ shown in the supplementary file). Hence, we performed a standardization (also called z‐normalization) of the variables.

We used the *standard Scalar* function of the *sklearn* to standardize the dataset. Specifically, to prevent information leakage about the test dataset into the model, we fitted the *standard scalar* using the training dataset. We used this information to standardize the test dataset.

We compared the performance of 6 machine learning models ‐Support Vector classifier (SVC), random forest classifier (RFC), logistical regression (LR), gradient boosting classifier (GBC), multilayer perceptron (MLP), and stacking classifier (SC). We tuned the hyperparameters of SVC, RFC, GBC, and LR using a five‐fold cross‐validation for the grid search strategy (we used 10‐fold cross validation for the analysis on the training dataset) that allows for an exhaustive search over the specified grid of parameters. Table 1 shows the values of specific parameters used and the value of tuned parameters. Stacking classifier is an ensemble‐based learning technique where the predictions of multiple classifiers are used as new features to train a classifier. We used Random forest predictions and Gradient boosting to train a Support vector classifier.^12^, ^17^, ^18^, ^19^ To avoid overfitting, we used a stratified 10‐fold cross‐validation strategy on the training dataset for each model. Stratified K‐fold cross‐validation (k = 10 in this case) is an approach where the training data set is split into multiple small sets, and the algorithm is trained on K‐1 sets and used on the remaining set for validation. For each fold, we used precision, recall, and f1‐score as the metrics to evaluate the performance of the models. Finally, the model was applied to the testing dataset. For each model, we computed the confusion matrix for both the training and testing dataset.

**TABLE 1:** The value of the tuned parameters used for the machine learning algorithms

Algorithm	Feature space	Tuned model
SVR	‘C’ = [0.01,0.02,0.03,0.04,0.05,0.005]	SVC(C = 0.03,break_ties = False,cache_size = 200,class_weight = None,coef0 = 0.0,decision_function_shape = ‘ovr’,degree = 3,gamma = ‘auto’,kernel = ‘rbf’,max_iter = ‐1,probability = True,random_state = 42,shrinking = True,tol = 0.001,verbose = False)
RFC	‘min_samples_leaf’: [1,2,3,4,5], ‘min_samples_split’: [2,3,4,5], ‘n_estimators’: [80,100,120],	RandomForestClassifier(bootstrap = True, ccp_alpha = 0.0,class_weight = None,criterion = ‘entropy’,max_depth = 4,max_features = ‘auto’,max_leaf_nodes = None, max_samples = None,min_impurity_decrease = 0.0,min_impurity_split = None,min_samples_leaf = 4,min_samples_split = 2,min_weight_fraction_leaf = 0.0,n_estimators = 100, n_jobs = None,oob_score = False,random_state = 42, verbose = 0,warm_start = False)
GBC	‘learning_rate’:[0.01,0.001,0.0001], ‘n_estimators’:[80,100,120], ‘min_samples_split’:[1,2,3,4,5], ‘min_samples_leaf’:[2,3,4,5],	GradientBoostingClassifier(ccp_alpha = 0.0,criterion = ‘friedman_mse’,init = None,learning_rate = 0.01,loss = ‘deviance’,max_depth = 4,max_features = ‘auto’,max_leaf_nodes = None, min_impurity_decrease = 0.0, min_impurity_split = None,min_samples_leaf = 4,min_samples_split = 2,min_weight_fraction_leaf = 0.0,n_estimators = 120,n_iter_no_change = None,presort = ‘deprecated’,random_state = 42,subsample = 1.0,tol = 0.0001,validation_fraction = 0.1,verbose = 0,warm_start = False)
LR	‘penalty’ : [‘l1’, ‘l2’], ‘C’:[0.1,1,5,10,50,100,1000]	LogisticRegression(C = 0.1,class_weight = None,dual = False,fit_intercept = True,intercept_scaling = 1,l1_ratio = None,max_iter = 1000,multi_class = ‘auto’,n_jobs = None,penalty = ‘l2’,random_state = 42,solver = ‘lbfgs’,tol = 0.0001,verbose = 0,warm_start = False)

Additionally, we plotted the receiver operating characteristic (ROC) Area under the curve (AUC) for each machine learning model. For the Random forest classifier, we computed the importance of each feature in classifying the albuminuria. Our analysis was conducted in python version 3.6 (https://www.python.org) using the library Scikit Learn.^20^, ^21^, ^22^ Python code for the entire processing pipeline is stored in the GitHub repository (https://github.com/prasu2172/Albuminuria).

## 3 RESULTS

**FIGURE 1:**
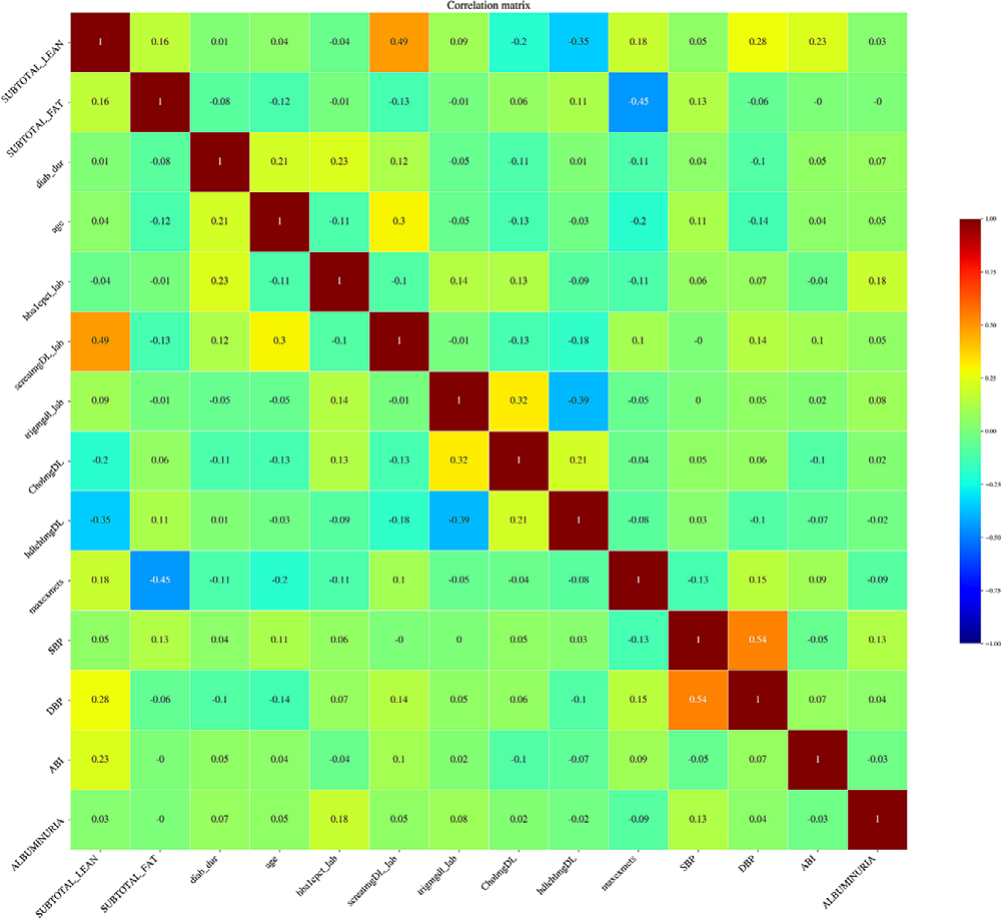
The correlation matrix after removing the highly correlated variables of body composition

**FIGURE 2:**
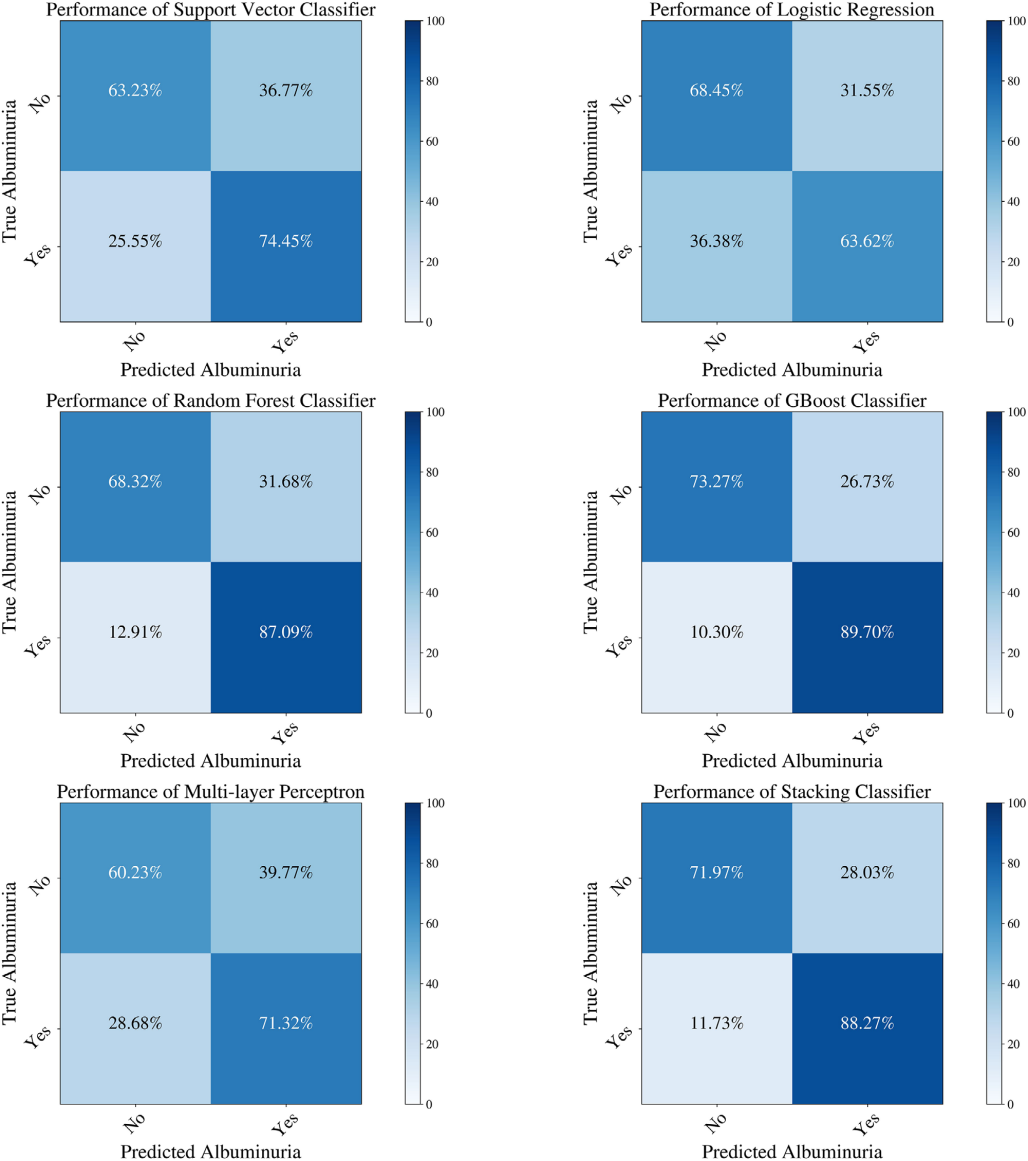
The confusion matrices of the different machine learning models in the training dataset

**FIGURE 3:**
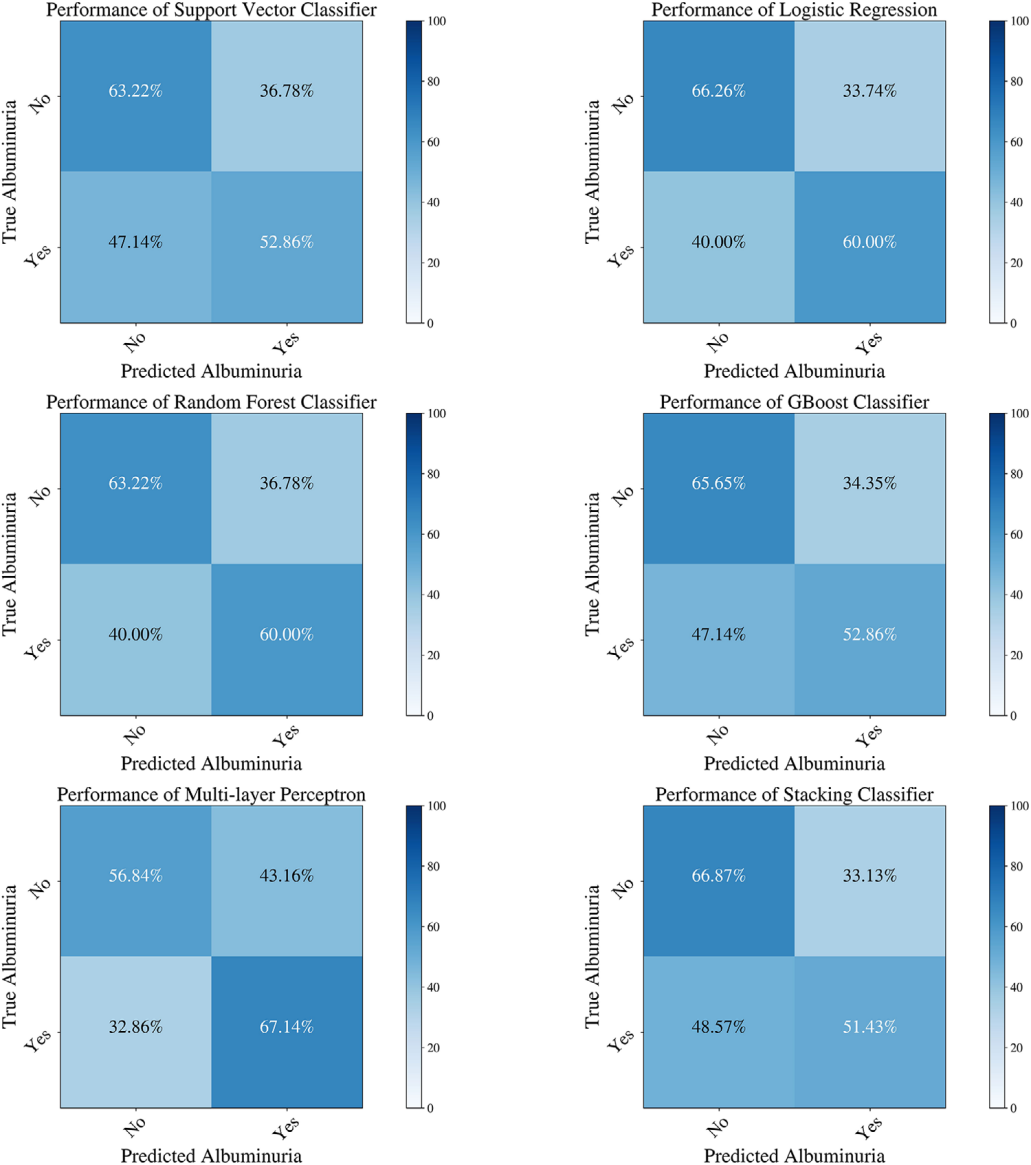
The confusion matrices of the different machine learning models in the testing dataset

**FIGURE 4:**
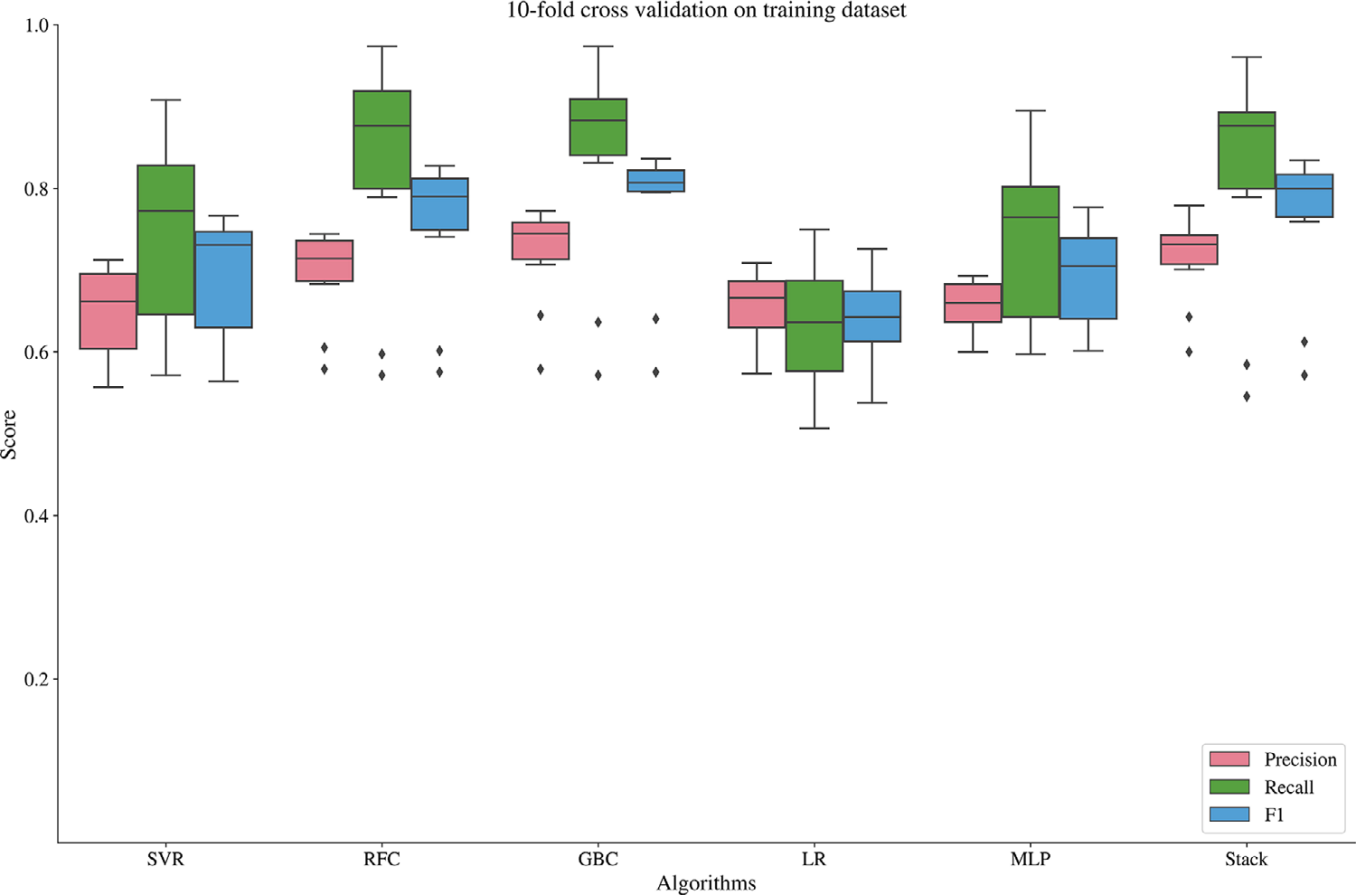
The results of the cross‐validation of the different models

Table 2 shows the descriptive statistics of the different features used in the model. Some body composition metrics had a strong correlation with one another and, as mentioned above, were removed from the final analysis. Figure 1 shows a pairwise correlation matrix after removing the correlated features. The confusion matrices for the different models in the training dataset Figure 2. All the models showed excellent precision, recall as well as F1 scores in the training dataset. The confusion matrices for the testing data are shown in Figure 3. Figure 4 shows the results of cross‐validation.

**TABLE 2:** Descriptive statistics of over 1300 participants showing the different factors and their distribution

Parameter	Mean	SD	Min	25%	50%	75%	Max
Subtotal Lean(g)	50 618.84	10 026.94	28 947.80	42 762.02	49 439.23	57 956.04	80 409.82
Subtotal Fat (g)	38 873.46	10 400.89	17 980.00	31 008.20	36 900.25	45 606.53	72 435.67
Diabetes Duration (years)	6.63	6.20	0.00	2.00	5.00	10.00	46.00
Age (years)	58.38	6.59	45.00	55.00	58.00	63.00	75.00
A1C (%)	7.31	1.21	4.70	6.40	7.10	7.98	12.50
Serum Creatinine (mg/dL)	0.80	0.20	0.40	0.70	0.80	0.90	1.80
Serum Triglycerides(mg/dL)	194.16	131.48	21.00	115.00	165.00	233.75	1527.00
Total Cholesterol(mg/dL)	194.43	37.07	82.00	167.00	192.00	217.00	405.00
HDL Cholesterol (mg/dL)	43.40	11.63	15.00	35.00	42.00	50.00	112.00
Maximum Exercise Capacity (Mets)	7.47	1.94	3.70	6.00	7.15	8.70	15.30
Systolic Blood Pressure(mmHg)	129.85	17.22	77.00	117.00	129.00	141.50	209.50
Diastolic Blood Pressure(mmHg)	69.85	9.41	42.50	63.50	70.00	76.50	100.00
Ankle Brachial Index(ratio)	1.17	0.14	0.67	1.08	1.16	1.24	2.68
Albumin to Creatinine Ratio	0.18	0.38	0.00	0.00	0.00	0.00	1.00

**FIGURE 5:**
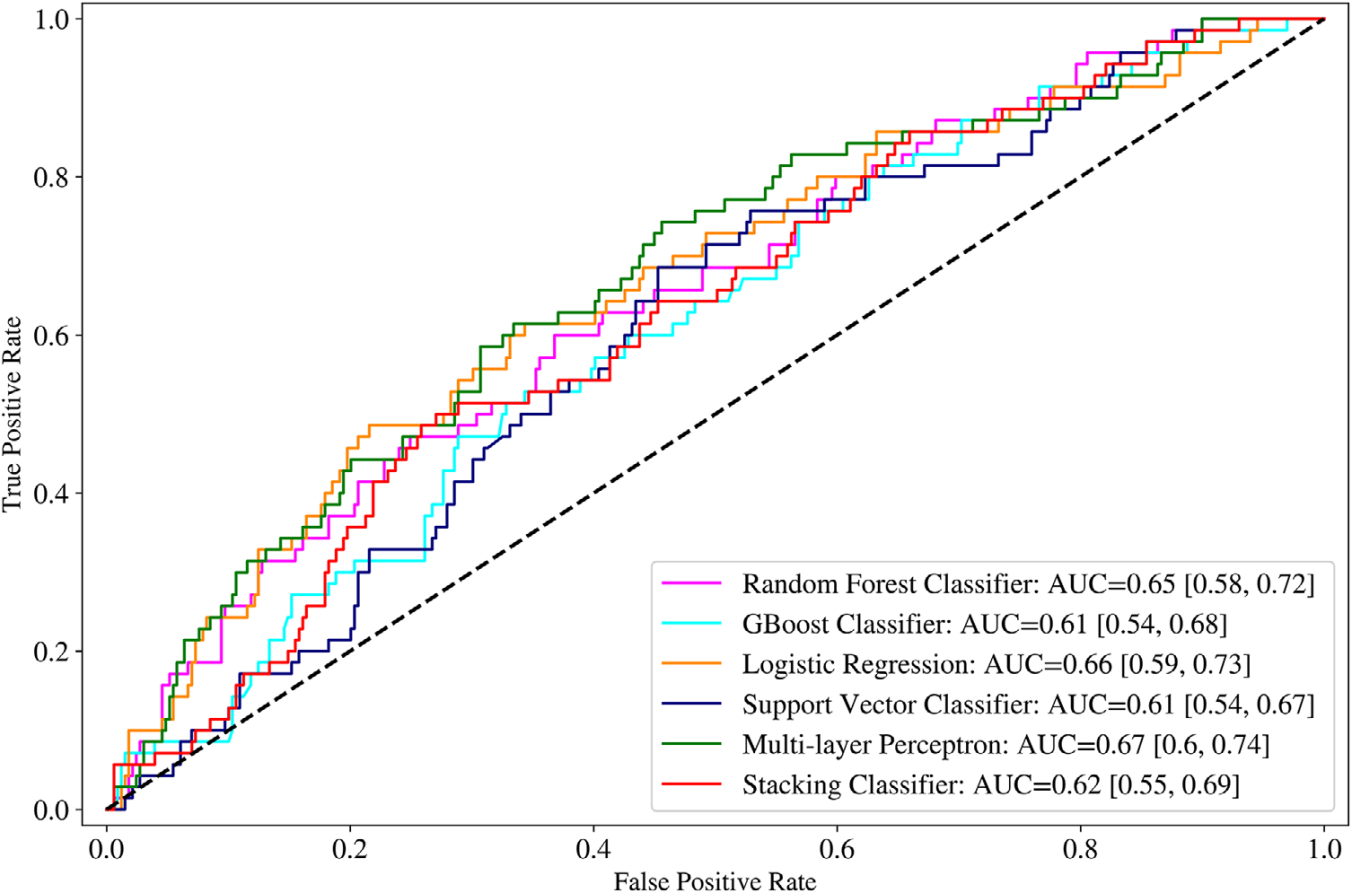
The ROC curves of the different models showing the Area Under the Curves (AUCs)

**FIGURE 6:**
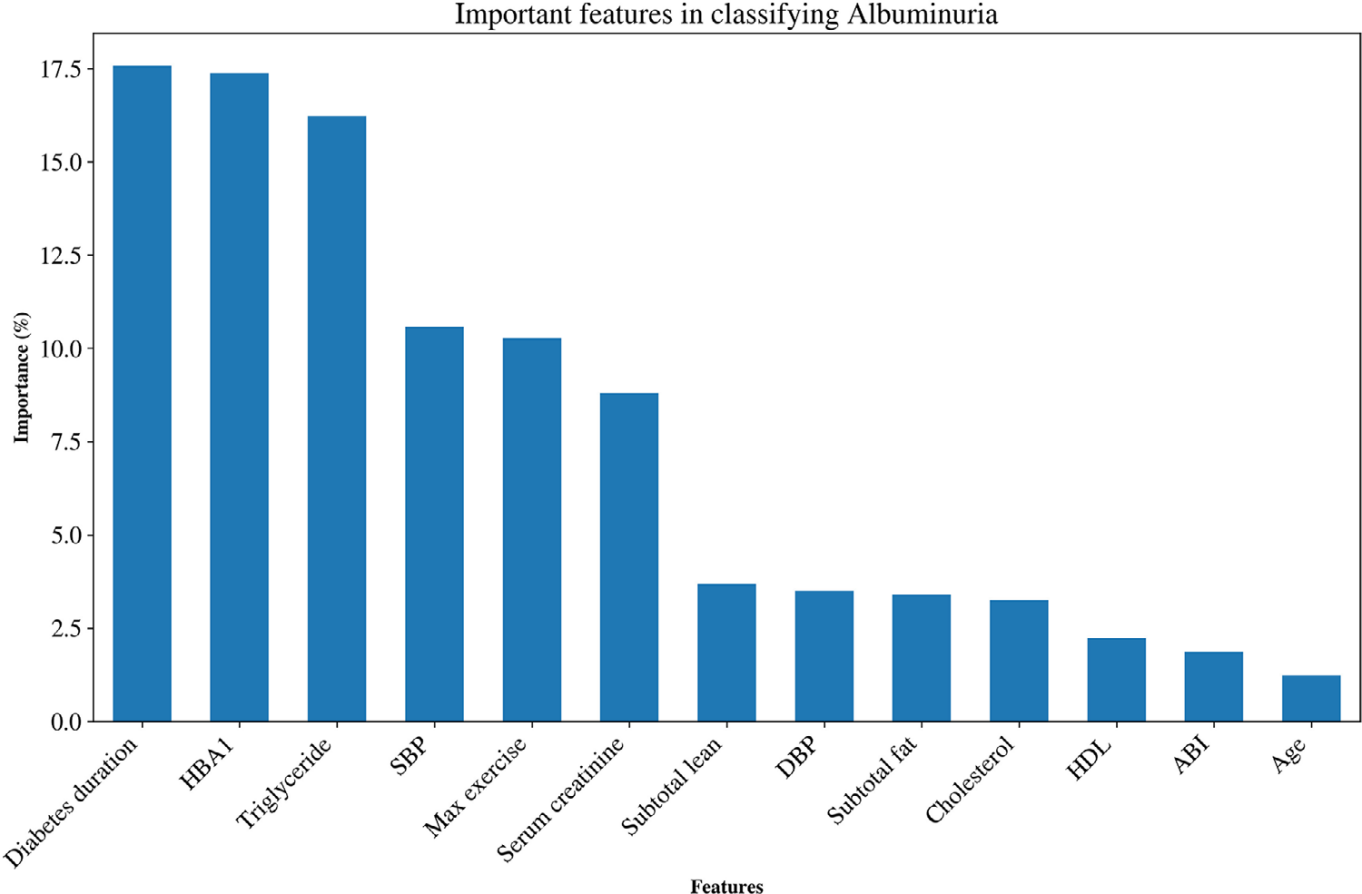
The feature selection based on the level of importance (based on the Random Forest Classifier)

The ROC curves are shown in Figure 5. The different models' AUC was as follows: Random forest classifier‐0.65, gradient boost classifier‐0.61, logistic regression‐0.66, support vector classifier ‐0.61, multilayer perceptron ‐0.67, and stacking classifier‐0.62. The essential features for classifying albuminuria are the duration of diabetes, A1C, serum triglycerides, SBP, Maximum exercise Capacity, serum creatinine, subtotal lean mass, DBP, and subtotal fat mass (in that order). The feature selection based on the level of importance (based on the random forest algorithm) is shown in Figure 6.

## 4 DISCUSSION

Our study shows that machine learning algorithms can help enhance our understanding of the determinants of albuminuria in persons with diabetes. The purpose of our study was to evaluate the strength of the different predictors that determine the presence of albuminuria in diabetes. Our study does show that machine learning algorithms help enhance our understanding of the determinants of albuminuria in persons with diabetes, compared to traditional statistics methods like logistic regression. The study also confirmed that the duration of diabetes, hemoglobin A1C, serum triglycerides, systolic blood pressure, and exercise capacity are the most important predictors of albuminuria, based on the feature selection. It reaffirms that controlling diabetes and blood pressure and maintaining body fat might delay the development of albuminuria. The study shows that body composition obtained through DXA scan might offer significant insight into the metabolic health in persons with diabetes.

Urinary albumin excretion is an established risk factor for the prediction of poor metabolic health. In a research paper from the Framingham heart study cohort, low albumin level(s) in the urine (less than 30 mcg) was associated with increased risk of cardiovascular disease and death, even after adjustment of other important risk factors in the nondiabetic nonhypertensive population.^23^ Albuminuria is strongly associated with calcification within the coronary and carotid arteries in Caucasians with type 2 diabetes, even if renal function is preserved.^24^

Prior studies have shown that sarcopenia, obesity are all associated with albuminuria in persons with diabetes.^25^, ^26^ In a study using the LOOK AHEAD cohort, different predictors like age, sex, race, duration of diabetes, A1C, hypertension, and ace inhibitors administration were used in a multivariate logistic regression model to examine the risk of albuminuria and obesity.^26^ The highest quartile of BMI was associated with albuminuria.^26^ However, the study only examined the relationship between total body fat percent and albuminuria, and it did not find an association between the two.^26^

Central obesity in nondiabetics is an independent predictor of albuminuria in South Asian subjects.^27^ This phenotype, in particular, can explain the higher incidence and poor outcome of microvascular complications like diabetic nephropathy in this population.^28^, ^29^ Furthermore, this observation points to the impact of central obesity and insulin resistance(that predates the onset of overt diabetes) in the development and progression of kidney disease due to an increase in oxidative stress and inflammation.^30^ In our study, subtotal lean and fat mass were associated with albuminuria(after removing other highly correlated body composition metrics).

There are some limitations to our studies. Our model can give an understanding of the development of albuminuria without giving an accurate prediction. This might be related to other known or unknown factors not being included in the analysis. For example, medication use, has not been incorporated into the analysis. We have not accounted for the use of Angiotensin‐Converting Enzyme (ACE) inhibitors, other antihypertensives, and antilipidemic agents. Also, the advent of new therapies like SGLT2 inhibitors might substantially alter the landscape of albuminuria in diabetes. Nevertheless, the study offers some critical insights into the role of exercise and body composition in determining albuminuria.

In summary, when applied to metabolic imaging (in the form of a DXA scan), machine learning techniques may offer unique insights into the risk factors that determine the development of albuminuria in diabetes.

## GENERAL NIDDK REPOSITORY ACKNOWLEDGMENTS

The Look AHEAD study was conducted by the Look AHEAD Investigators and supported by the National Institute of Diabetes and Digestive and Kidney Diseases (NIDDK). The data and samples from Look AHEAD reported here were supplied by the NIDDK Central Repositories. This manuscript was not prepared in collaboration with Investigators of the Look AHEAD study and does not necessarily reflect the opinions or views of the Look AHEAD study, the NIDDK Central Repositories, or the NIDDK.

The data was provided to us in accordance with the NIDDK‐NIH researcher data sharing agreement.

## CONFLICT OF INTEREST

The authors of the manuscript have no disclosures to make and report no conflict of interest.

## PATIENT CONSENT STATEMENT

The work was conducted on the de‐identified data obtain from the NIH central repository as outlined below in the acknowledgement section. Informed consent from the participants was obtained by the original LOOK AHEAD STUDY Group.

## AUTHOR CONTRIBUTIONS

Design and concept; Zeid Khitan, Prasanna Santhanam, Tanmay Nath

Data and study compilation; Tanmay Nath, Prasanna Santhanam

Manuscript preparation; Zeid Khitan, Tanmay Nath, Prasanna Santhanam
